# Part I: cancer in Sudan—burden, distribution, and trends breast, gynecological, and prostate cancers

**DOI:** 10.1002/cam4.378

**Published:** 2015-01-30

**Authors:** Amany Elamin, Muntaser E Ibrahim, Dafalla Abuidris, Kamal Eldin H Mohamed, Sulma Ibrahim Mohammed

**Affiliations:** 1 Department of Comparative Pathobiology, Purdue UniversityWest Lafayette, Indiana, 47907; 2 Purdue University Center for Cancer ResearchWest Lafayette, Indiana, 47907; 3 Commission for Biotechnology and Genetic Engineering, National Center for ResearchKhartoum, Sudan; 4 Department of Molecular Biology, Institute of Endemic Diseases, University of KhartoumKhartoum, Sudan; 5 National Cancer Institute, University of GeziraWadmadani, Sudan; 6 Radiation and Isotopes Center Khartoum (RICK)Khartoum, Sudan

**Keywords:** Africa, cancer, sub-Saharan, Sudan

## Abstract

Despite the growing burden of cancer worldwide, it continues to receive low priority in Africa, across the continent and specifically in Sudan. This is due to political unrest, limited health resources, and other pressing public health issues such as infectious diseases. Lack of awareness about the magnitude of the current and future cancer burden among policy makers play a major role as well. Although, the real scope of cancer in Sudan is not known, the reported cases have increased from 303 in 1967–6303 in 2010. According to Globocan estimates, the top most common cancers in both sexes are breast, non-Hodgkin lymphoma, leukemia, esophagus, and colorectum. This review is the first of four papers that focuses on cancer, its distribution and trend as well as the risk factors most common in Sudan. It is expected that cancer will increase in Sudan as a result of migration of people from rural areas to urban cities in the pursuit of a better standard of living, which has resulted in lifestyle and behavioral changes that include tobacco chewing and smoking, unhealthy dieting, and a lack of physical activity. These changes are further exacerbated by the aging population and have made the country vulnerable to many diseases including cancer. These reviews are meant to provide a better understanding and knowledge required to plan appropriate cancer-control and prevention strategies in the country.

## Introduction

Cancer is a leading cause of death worldwide. An estimated 12.7 million new cancer cases occurred in 2008, of which about 715,000 new cancer cases resulted in 542,000 deaths in Africa 1. These numbers are projected to nearly double to 1.28 million new cancer cases and 970,000 cancer deaths by the year 2030. This increase in cancer cases in Africa is attributed to both aging and population growth, and adoption of lifestyles associated with economic development, such as smoking, unhealthy dieting, and a lack of physical activity 1,2.

Cancer continues to receive low public health priority in Africa, in general across the continent and specifically in Sudan. This is due to the limited health resources and other pressing public health issues such as malaria, tuberculosis, and acquired immune deficiency syndrome/human immunodeficiency virus 3. Lack of awareness about the magnitude of the current and future cancer burden among policy makers and the public in Sudan play a critical part as well.

Sudan was the largest country in Africa until 2011, when South Sudan separated into an independent country. Officially is called the Republic of Sudan. Sudan is bordered by Ethiopia to the east, Kenya to the southeast, Uganda to the south, the Democratic Republic of the Congo to the southwest, the Central African Republic to the west, and Egypt to the north. Currently, its total area is about one million square miles. The Sudanese population, 34,206,710 million, is highly diverse, consisting of about 19 different ethnic groups and almost 600 subgroups. Sudanese Arab form approximately 70% of the population, with Fur, Beja, Nuba, and Fallata make the rest of the population 4,5. Its diverse ethnic populations emigrated from the surrounding regions and various regional climates. More than 80% of the Sudanese population lives in rural areas or are nomadic which present a great challenge to any disease control initiative 6. On average 46.5% of Sudan population is under the poverty line ranging from 25% in Khartoum to 75% in Northern Darfur 3. Youth (15–24 years old) unemployment exceeds 25.4%. However, 62% of the population (37,195,000) 15 years and older in Sudan are literate, of those 79% live in urban areas while 51% of rural origin 3. Most health providers reside and work in Khartoum State, limiting the ministry of health capability to provide healthcare services to people living in rural areas of Sudan 7.

Political and economic instabilities in Sudan led to inefficiency and inadequacy of health system. Until recently, cancer in Sudan was a relatively unknown health problem with most public health efforts directed toward tropical and infectious diseases as mentioned above. Now, cancer incidence has been growing at an average annual rate of 0.061 over the last five decades 1967–2010 and is likely to continue to grow (See Table1, Ref, 8). According to Globocan estimates, the top most common cancers in both sexes are breast, non-Hodgkin lymphoma, leukemia, esophagus, and colorectum 2.

This review attempts to scrutinize most if not all cancer studies published to date in Sudan, and highlight their most significant findings in term of cancer burden, pattern, genetic, and environmental causes of cancer. It also expected to lead to a better understanding of the current status of cancer burden, distribution, and trend as well as cancer research in Sudan.

**Table 1:** Cancer frequencies in Sudan 1967–2010; adopted from Mohammed et al., 2013^8^

Year	Number of incidents of cancer	Cancer rate/1000	Year	Number of incidents of cancer	Cancer rate/1000
1967	303	0.0234	1989	1357	0.0558
1968	448	0.0339	1990	1572	0.0629
1969	540	0.0400	1919	1494	0.0582
1970	512	0.0371	1992	2157	0.0817
1971	538	0.0382	1993	1847	0.0722
1972	500	0.0348	1994	1645	0.0625
1973	562	0.0398	1995	1733	0.0640
1974	692	0.0472	1996	1810	0.0649
1975	470	0.0308	1997	2119	0.0739
1976	565	0.0357	1998	2145	0.0727
1977	738	0.0449	1999	2102	0.0692
1978	545	0.0319	2000	2541	0.0813
1979	568	0.0320	2001	2963	0.0922
1980	704	0.0381	2002	3070	0.0928
1981	672	0.0350	2003	3185	0.0936
1982	773	0.0388	2004	3450	0.0986
1983	870	0.0422	2005	3705	0.1029
1984	913	0.0431	2006	3505	0.0946
1985	903	0.0415	2007	4813	0.1262
1986	1112	0.0497	2008	5156	0.1317
1987	927	0.0403	2009	5739	0.1425
1988	1308	0.0553	2010	6303	0.1522

## Methods

We have searched the U.S. National Library of Medicine literature search engine (PubMed), Google scholar, Google, and Sudan Medical journal using terms including “Sudan” and “cancer, breast, gynecological malignances, cervical cancer, and prostate.” We have not imposed any dates restriction, as our purpose is to review all the literature about cancer in Sudan and to prepare a comprehensive report. This search resulted in many publications covering topic in all aspects of cancer research including epidemiology, diagnosis, clinical presentation, and treatment.

## Cancer in Sudan

**Figure 1:**
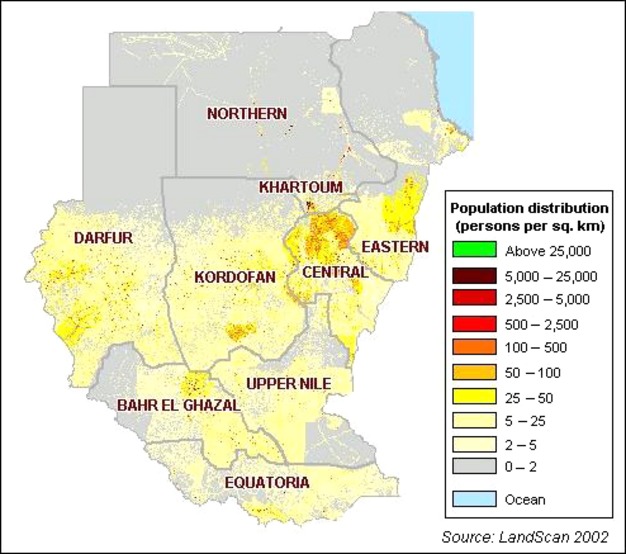
Sudan population density map adopted from LandScan 2002.

Sudan is divided into 26 states and districts with varying number of population densities (Fig.1). States' Ministries of Health, Armed forces, Universities, Police, and private sector collectively, in an uncoordinated manner, provide health services to the people of Sudan. The public sector health services in Sudan are organized at three levels primary, secondary, and tertiary. The states' general hospitals are the referral centers for the entire state. Specialized centers and Khartoum General Hospital, located in capital Khartoum, constitute the tertiary level (Federal ministry of health web site). Radiation and Isotope Center at Khartoum (RICK) and National Cancer Institute at Gezira University (NCI-GU) are the only two specialized cancer centers providing chemotherapy and radiotherapy services for all 26 states. After exhausting all the medical attempts for treatment at the primary and secondary care facilities as well as local healers, patients are referred to RICK or NCI-GU depend on the proximity to the patient's resident.

Till recently, the majority of published reports on cancer data in Sudan were retrospective descriptive hospital-based studies. There is no National Cancer Registry (NCR) in Sudan. Lack of basic health services (diagnostic and treatment facilities), stability of the population, individual identity, trained personal, follow-up, and nonavailability of census data and death certificates contributed to the lack of accurate figures about the true incidence of cancer in Sudan. Therefore, most publications reported absolute numbers or frequency ratios of tumors.

Although, the first NCR in Sudan started in 1967 with a grant from the International Union against Cancer, it was short lived and its activity was discontinued in early 1980s due to lack of funds. At that time, the main sources for cancer data are the Department of Pathology at University of Khartoum Faculty of Medicine, and the Stack Medical Research Laboratory of the Ministry of Health, currently known as the National Health Laboratories (NHL). The data from these two institutions are based on histopathologically confirmed cases. Since 1978, the NHL provided histopathology services for the entire Sudan, which aided in accruing a cancer database within the Center 9,10.

Another source of cancer data is RICK, a hospital-based registry. Until recently, RICK was the only center specialized in radiotherapy treatment throughout Sudan. RICK started in 1964 as a small laboratory at Khartoum Specialized Teaching Hospital. In cooperation with International Atomic Energy Agency, RICK was officially inaugurated in December 1966 as a separate hospital. At its humble beginning RICK mainly treated cancers as well as diagnosed the disease using radioactive isotopes. Early in the 1980s, the center expanded to include nuclear as well as clinical departments with all the necessary cancer expertize including clinical radiotherapists, medical oncologists, pediatric oncologists, nuclear medicine specialists, diagnostic radiologists, and patient's social and psychological services. The center receives referrals from all over the country. Two senior clinical oncologists and twenty junior consultants, one medical oncologist, and two pediatric oncologists operate the center. As of 2005, the center sees about 5000 cancer patients per year. Data were collected manually before 1999, since then they have been computerized using Statistical Product and Service Solution (SPSS) software package (IBM, Inc. Armonk, NY) 9.

Thereafter, more additional regional histopathology laboratories were established. The first was established at the University of Gezira in 1979. Later in 2006, the University of Gezira with the support of the International Agency for Research (IARC) established the first population-based cancer registry in the Sudan. It uses the CanReg4 format 9.

Recently, in 2009 with funding from the Ministry of Health, a population-based NCR was established in Khartoum. The Ministry of health's plan was to create a cancer institute in all of the 14 States within which a regional cancer registry would be established. The registry is staffed with a director, data collection, and entry personnel. The NCR is charged with developing a system that will facilitate creation and maintenance of the local and regional data and merging the data into a central accessible system 9,11.

The first report about cancer in Sudan, “Malignant epithelial tumors in the Sudanese,” was published by Hickey in 1959 following a lecture presented to the Royal College of Surgeons, England on 13 March 1958 12. The report presented data on 1335 malignant epithelial neoplasms histopathologically diagnosed at the Stack Medical Research Laboratories (NHL) from 1935–1954. At that time, the most common tumor sites were the skin (32.8%) followed by the breast (22.9%). Following that period (1954 = 61), a report on data from NHL and the Department of Pathology, University of Khartoum showed that the number of cancer cases almost doubled to 2234 malignant neoplasms 11. In Khartoum district alone, 1578 malignant tumors were recorded during the period of 1957 to 1965 at the Department of Pathology, which mainly serves Khartoum Civil Hospital 13. In 1976, Malik and colleagues 14 reported on 8212 cases received at the University Department of Pathology and the NHL during 1962–1973. Superficial cancers (squamous carcinoma, basal carcinoma, malignant melanoma and Kaposi's sarcoma) constituted the majority of the cases accounting for 16.8% (1379/8212) of all cancers followed by the breast (11.8%; 937/8212). In addition, in 1978, 1036 malignant tumors were recorded at the Sudan Cancer Registry at NHL 15. Breast cancer was the most common cancer and account for 13.7% followed by skin 10.3%. Furthermore, during 1967–1984, 10,410 cancer patients were seen at RICK 16. Most male patients presented with cancer of nasopharynx, non-Hodgkin lymphoma, mouth cancers (gingiva), and carcinoma of the urinary bladder and Kaposi sarcoma. While, female patients were commonly seen, in order of high frequencies, for breast, cervix, ovary, and mouth cancers 16. Obviously, there is increase in cancer number from 1957 to 1984 in Sudan. And only one report per every 10 years was published to document this increase. After 1984, most available published report focused on data from one single cancer.

In a recent, study from Sudan NCR. 6771 incident cancer cases were recorded among Khartoum State residents during 2009–2010 period. The age-standardized rates using the 1966 and 200 World Standard populations were 165.0 and 181.0 per 100,000 population. Of those, 3646 (53.8%) cases were women and 3125 (46.2%) were men. The incidence rate of breast cancer (25.1 per 100,000) was substantially higher than the other primary cancer sites. The most common primary cancer sites in women were breast, leukemia, cervix, ovary, lymphoma, esophagus, and colorectal cancer. In men, the most common cancer sites were prostate, leukemia, lymphoma, oral, colorectal, and liver 17.

Basic as well as clinical research is limited in Sudan and many reasons could be cited but mainly a lack of realization by governmental and private sectors of the importance of research and designating a budget for academic institutions and others to access as well as instituting a reward system for those who conduct research. Because of this, very few studies so far have attempted to determine cancer etiology in Sudan.

Out of those very few, one study was supported by the government and examined the level of radiation in the Northern State, where the recently established Sudan NCR reported the highest rates of cancer prevalence in the country. However, no abnormal increase in the levels of radioactive elements (238U, 232TH, 40K, and 137Cs) in the soil or air samples was observed. Comparing the radioactive levels of these elements with similar data from different regions in Sudan, worldwide, and from well-recognized high natural radiation background areas the Northern State's radioactive levels fell within the normal limits 18. Another study examined the possible association between cancer and environmental or social factors relevant to certain areas of central Sudan. Using the Geographic Information System and data from 1999–2008, the study found that over the past 10 years cancer rates increased primarily in some localities where fertilizers and pesticides were extensively used in Gezira agriculture scheme 19. Few studies have reported on cancer risk factors from nutritional aspects and habitual behavior such as toombak dipping, which is rich in carcinogenic nitrosamine. Furthermore, molecular studies on the etiology of cancer were very scarce in Sudan. Few publications reported on cancer association with tumor suppressor genes, oncogenes, and tumor viruses' infection 20.

A major challenge to the treatment of cancer in Sudan, as in most developing countries is that majority of the patients present with advanced stage disease. Seventy-eight percent of Sudanese cancer patients have stage III or IV disease at first presentation. In these advanced stages attempted treatment typically include surgery, radiotherapy, and chemotherapy and hormone therapy. Unfortunately, treatment of advanced disease has limited success. Many cancers such as cervical cancer are largely curable if detected early. However, there are no national cancer prevention programs adopted in the country to render cancer treatment more effective 6.

Another challenge to cancer control in Sudan is lack of domestic institutional collaborations and funds for cancer research in Sudan. In addition to loss of a great portion of data from a number of master degree dissertations and PhD thesis with outstanding findings that are not published and only kept in university libraries. This is because graduate students as well as faculty promotions are not conditional on publications and knowledge disseminations. Lack of research funds and hard currencies to cover publication fees in reputable international journals are another factors.

## Breast Cancer

Breast cancer continues to be the most common cancer among women in Sudan. In 1959, breast cancer comprised 22.9% of all cancers (*n* = 1335) followed by cancers of the genital tract. Majority of female patients were of menopausal age and the clinical course of the disease at that time appeared similar to the disease in European women 11. However, a recent study by Awadelkarim and colleagues highlighted possible differences between breast cancer in Sudanese women living in Central Sudan and women in Northern Italy. The Sudanese patients were premenopausal in age and presented with large tumors at more advanced stages and grades and frequently positive for nodal metastases compared to Italian patients. Estrogen receptors expression varied between the two groups with most Sudanese patients' tumors expressing no receptors 21. These clinicopathological and patient characteristics are now common throughout Sudan.

**Figure 2:**
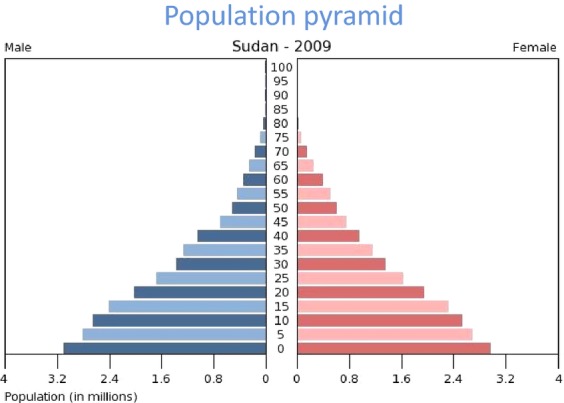
Sudan Population Pyramid 2009 (US Census Bureau).

Among breast cancer patients (*n* = 521) who visited Khartoum Teaching Hospital during a 5-year period from 1994 to 1999, invasive ductal carcinoma was the most common type (71.5%) and most patients (17.2%) had an advanced stage III and IV disease 22. Furthermore, among breast cancer patients (*n* = 1255) attending NCI-UG from 1999 to 2006, invasive ductal carcinoma was the most common type (82%). And about 74% of those patients were less than 50 years old and presented with stage III and higher tumors expressing no estrogen or progesterone receptors that had already metastasized 23. This indicates that women in Sudan are inflicted with breast cancer at young age. However, the mean age of breast cancer in Sudan and the entire Africa is younger compared to other developed countries because Africa including Sudan has the youngest population on the world (Fig.2). The majority of the tumors were invasive ductal carcinoma that lacked hormonal receptors expressions, and present with advanced disease.

The frequency of advanced breast cancer among women presenting with palpable lesions were very high in Sudan. Women patients who referred to a cytodiagnosis center in Khartoum (*n* = 200) with palpable breast lumps during a period of a year, 68 (34%) were diagnosed with malignant disease while 56 cases (28%) were fibroadenoma, 23 cases (11.5%) were fibrocystic change, 22 cases (11%) were inflammatory lesions (including mastitis and abscess formation), 12 cases (6%) were benign cysts, and the remaining 19 cases (9.5%) were with lactation changes, lipoma, gynecomastia, and phyllodes tumor 24.

### Breast cancer risk factor

Breast cancer risk factors among Sudanese patients (*n* = 150) and healthy control (*n* = 100) included past history of benign breast disease (*P < *0.04), previous breast biopsies (*P < *0.07), pesticide and plasticizer exposure (*P < *0.01), periods of being overweight (*P < *0.001), physical inactivity (*P < *0.0001), being unmarried (*P < *0.002), and decreased number of children (*P* < 0.002) 25. Breast cancer and other cancers in general are noted to be higher in the north than in other states of the Sudan according to cancer registry data at RICK. However, no significant differences (*P < *0.05) in term of demographical, hormonal, and family history-related risk factors between women living in the Northern States compared to women from other states treated at RICK. Therefore, this excludes living in north Sudan as a risk factor 26.

Recently, genetic and genomic risk factors associated with the development of breast cancer in Sudanese women were examined. BRCA2 exon 11, breast cancer susceptibility genes, and the conserved p53 regions were studied in breast cancer samples from 20 patients. One somatic mutation and one polymorphism in BRCA2 exon 11 and no mutation for all p53 sequences were found indicating a limited role of these regions in the pathogenesis of breast cancer in those patients 27. However, a study that screened for germline BRCA1/2 mutations in patients (*N* = 35) from Central Sudan detected a total of 60 sequence variants (32 in BRCA1, 28 in BRCA2) in 94% of the cases and five truncating mutations (2 in BRCA1, 3 in BRCA2) in 14% of the patients 28. Furthermore, *BRCA1* and *BRCA2* mutations were tested in female teenage students (*n* = 47) attending Marawi Secondary School in Northern State. About 51% of the students with a family history of breast cancer and 20% with no family history of breast cancer had *BRCA1* and *BRCA2* mutations. Most of the BRCA1 mutations located to exon 11 fragments 11.9 and 11.1 29. Though, in a study that examined methylation status of six tumor suppressor genes that included *BRCA1*, *BRCA2*, *p14*, *p16*, *hMLH,* and *MGMT* in breast (*n* = 23), other tumor (*n* = 10), and control tissues (*n* = 4) suggested that *BRCA1*, *BRCA2*, and *p14* appeared to be under strong epigenetic silencing. *BRCA1*, *BRCA2*, and *p14* were strongly hypermethylated in 84%, 84%, and 81% of cancer tissues, respectively 30.

Genetic alterations in estrogen receptor alpha gene (ESR1) such as C325G single nucleotide polymorphism (SNP) are thought to play a role in predisposition to breast cancer. Genotyping C325G in ESR1 in breast cancer patients (*n* = 100) in comparison to healthy controls (*n* = 90) revealed a significant association of breast cancer risk in women 50 years and younger who had the C allele (OR: 2.28, 95% CI: 1.104.72) (*P* = 0.03) suggesting that polymorphism within the low penetrance ESR1 is associated with breast cancer susceptibility in young Sudanese women 31. Similarly, genetic alterations in human epidermal growth factor receptor (HER-2/neu) have been shown to induce breast cancer malignant transformation. The association of HER-2/neu Ile655Val polymorphism and risk of breast cancer in a Sudanese population were examined and found to be borderline significant. Women who are heterozygous Ile/Val carriers have higher risk of breast cancer. Both ESR1325C and HER-2/neu Ile655Val variants were suggested to jointly contribute to a higher risk of breast cancer 32.

### Breast cancer subtypes

The overall frequency of basal-like subtype (ER-/PgR-/Her-2/neu-/basal CK+) was only 10% of 113 cases examined at the Histopathology and Cytopathology Department at RICK. This frequency is low compared to breast cancer cases from East and West Africa but much higher than the frequencies reported for Caucasian and African-American women in the United States of America 33. Furthermore, in a previous study about 75% and 55% of breast cancer cases from women (*n* = 40) presented at Khartoum Teaching Hospital during the period of 2000–2001 were estrogen receptors-positive and progesterone receptors-negative, respectively 34.

Recently, few studies have attempted to identify biomarkers for breast cancer in Sudanese women. High levels of Se, Zn, and Cr elements in breast cancer cases (*N* = 40) compared to matched normal breasts (*N* = 40) were suggested as candidate markers for early detection of breast cancer 34. Preliminary proteomic examination of Sudanese breast cancer and normal tissues (*n* = 24) identified Peroxiredoxin V (PrdxV) protein as differentially expressed in tumor tissues. Immunohistochemistry analysis of tumor (*n* = 77) and control (*n* = 68) tissues revealed that PrdxV protein was not expressed in 88.3% of breast cancer and majority of control tissues. Loss of this protein was suggested as a tumor marker of population specificity 35.

## Gynecological Cancers

During 1954–1961, cervical cancer ranked second after breast cancer accounting for 15.7% of the total malignancies in Sudanese women seen at the Khartoum Civil Hospital and all district hospitals followed by ovarian cancer (6.4%) 13. Cervical cancer accounted for 8.2% of all cancer types and ranked second top in women (*n* = 26652) treated at both RICK and NCI-UG over a 6-year period (2000–2006) 36. It comprised 7.9% of neoplasm cases (*n* = 195) diagnosed during the period from 2004–2009 at the National Health Laboratory 37. Current estimates indicate that every year 833 Sudanese women are diagnosed with cervical cancer (estimated age-standardized incidence: 7.9 per 100,000 per year) and 534 die from the disease 38.

In Sudan, more than two-thirds of all women with invasive cervical cancer are diagnosed at an advanced stage. Mostly are older, and live in rural areas 36,37. The mean age of patients (*n* = 195) diagnosed with cervical cancer at the NHL during 2004–2009 is 53.25 years. Histopathologically, 95.9% of the cases were carcinomas (Squamous cell carcinomas, 90.9%, Adenocarcinomas, 4.8% and other epithelial tumors 4.3%). Of the Squamous carcinomas, 98.8% were invasive and 1.2% were intraepithelial neoplasia. Non-keratinizing squamous cell carcinomas and keratinizing squamous cell carcinomas accounted for 66.1% and 24.4%, respectively 38.

### Cervical cancer risk factors

The major risk factor for cervical cancer is human papilloma virus (HPV). However, few published studies explored this association in Sudanese women. In Northern Africa overall (including Sudan), an estimated 10.7% of women in the general population harbor cervical HPV genome and about and 78.4% of invasive cervical cancers were attributed to HPVs 16 or 18 39. In a study of 40 women with cervical cancer 16 (40%) were positive for HPV subtypes 16 and 18 36. HPV 16 and 18 were detected also at a frequency of 93.6% in cervical cancer samples in a similar study 40. In addition, similarity between Sudanese (94%) and Ethiopian (93%) women with cervical cancer in term of HPV prevalence was reported. HPV 16 was the most frequent genotype identified in samples from both countries (91%) and (82.5%), respectively 41.

Tobacco use was also found to significantly associate with cervical cancer in Sudanese women. A study of 100 women with cervical cancer at RICK and 100 healthy controls from Ahfad Reproductive Health Center (ARHC) in Omdurman during 2009–2010 revealed that 55% of the data data into a central accessible system previously 42.

The combined risk of the loss of heterozygosity of intron 1 and/or intron 17 in the retinoblastoma gene (63%) together with the high mutation frequency of the p53 codon 72 arginine allele (43.6%) indicate a possible epistatic effect of the two genes/polymorphisms in cervical carcinogenesis 40. A statistically significant association, after adjusting for age, educational level, employment, and potential confounding factors such as smoking, number of sexual partners, and use of contraceptive method was found between cervical cancer and the following risk factors including uterine cervix laceration (odds ratio [OR] 18.6; 95% confidence interval [CI]: 4.64–74.8), assisted vaginal delivery (OR 13.2; 95% CI: 2.95–54.9), parity (OR 5.78; 95% CI: 1.41–23.7), female genital mutilation (OR 4.78; 95% CI: 1.13–20.1), and episiotomy (OR 5.25; 95% CI: 1.15–23.8) 43.

### Cervical cancer screening in Sudan

A community-based survey of Sudanese women living in Khartoum, from 2003–2008, of whether they had Pap smear, reported that 35% (90/256) of the respondents had never had a Pap smear while 65% (166/256) had one test within the last 3 years. The study concluded that there is a low level of cervical cancer screening among Sudanese women, which reflects a lack of awareness and resources as fundamental factors that impede cervical cancer early detection in Sudan 44. It has been estimated that less than 0.10% of women in Sudan had been screened for cervical cancer in the last 5 years 42,45. In present day Sudan, there is still no adopted cervical cancer-screening program. The traditional Pap test is expensive, requires an orchestrated effort for evaluation, follow-up and treatment, as well as trained cytologists and pathologists. In place of the Pap test Ibrahim and colleagues examined the feasibility and acceptability of visual inspection with the acetic acid (VIA) method in a cross-sectional prospective pilot study of 100 asymptomatic women living in Khartoum State 43. About 16% of women screened had positive VIA test (i.e., indicative of a cervical lesion) demonstrating the VIA screening method to be feasible and acceptable to Sudanese participants 43. Similarly, another cross-sectional study of 934 asymptomatic women living in Khartoum conducted from 2009–2010 using VIA, indicated that VIA was useful for cervical cancer screening in the primary health care setting in Sudan 46,47. ISH HPV test reported to be more predictive of HPV infection among Sudanese women with gynecological complains (*n* = 106) than the Pap test. ISH HPV and Pap tests detected HR-HPV in 27.3% and 5.7% of cervical samples, respectively 48.

### Oncopsycological studies

Oncopsycological studies remained unexploited in cancer patients in Sudan. In fact, quality of life (QOL) issues in cancer care are rarely studied in developing countries, despite the rising numbers of breast and gynecologic cancers 49,47,50,51. It has been shown that Sudanese breast cancer patients who are in stable condition and having psychosocial support possessed hope and a good QOL. While caregivers (if female, parent, young, less educated, and unemployed) are generally vulnerable and need support than the cancer patient recently diagnosed with cancer, less educated, single, and unemployed 52. With regard to sexual health issues, Sudanese women with breast cancer experienced loss of sexual desire and satisfaction compared to healthy women controls and varied according to the type of cancer treatment received as well 53.

## Prostate Cancer

Prostate cancer is the most common cancer in Sudanese men 54. The age-standardized rate is 10.3 and mortality is 8.7 per 100,000 population. It ranked second among all cancers in both sexes after breast in 2012 2.

Three decades ago, prostate cancer ranked tenth among all men cancers diagnosed at the Sudan Cancer Registry in 1978, less frequent than skin cancers and non-Hodgkin lymphoma (*n* = 1036) 15. Moreover, prostate cancer represented only 0.8% of all male cancers (*n* = 10410) investigated at RICK (1967–84) 16. This recent increase in comparison to decades ago could have been due to progress in diagnostic techniques introduced lately in the country. As a result, prostate cancer was the most diagnosed cancer among men accounting for 7.6% of all cancer types in men (*n* = 10911) at both RICK and NCI-UG during year 2000–2006 55. Recently, prostate cancer was the most common cancer among male patients treated at the NCI-UG 56. It ranked first among cancer male patients (*n* = 268) treated in the NCI, central Sudan (2006–2009). The disease was found equally distributed among different tribes and most cases (85.4%) presented with stage III and IV. The mean age of patients was 72.2 ± 9.25 57.

### Prostate cancer Risk Factors

Despite the fact that prostate cancer ranked first among all cancers in Sudanese men, few publications described the epidemiology and pathology of the disease. The common potential risk factors for prostate cancer among patients (*n* = 268) referred to NCI, central Sudan during 2006–2009, included age, and history of tobacco and alcohol consumption. Family history was positive in only 6.7% of the patients and 73% smoked or consumed alcohol. The most common occupation risk factor was farming (60.1%) 57.

### Prostate cancer diagnosis

As stated above, the detection of prostate cancer improved after the introduction of effective diagnostic methods. Internationally, Prostate-Specific Antigen (PSA) Test levels between 4 and 10 ng/mL regarded as suspiciously abnormal. However, PSA value of 4 ng/mL (mean 1.85 ng/mL total) was reported in both men with prostatic adenocarcinoma (13.1%) and benign hyperplasia (86.7%). Therefore, the cutoff point for total PSA was lowered to 0.2–2.1 ng/mL (free PSA to total PSA ratio of 11–20%) for screening Sudanese men for prostate cancer 58. PSA mean of 1.48 ng/mL was reported among Sudanese men with no recent urinary tract infection (*n* = 1051), age 40–90 years, seen at the Central Laboratory Services, Soba University Hospital, Khartoum during 2008–2010 59.

The combination of digital rectal examination (DRE) and PSA increased prostate cancer detection rates more than PSA alone 58. DRE and PSA each had sensitivity of 63.8% and 91.6%, specificities of 68% and 24%, and positive predictive values (PPV) of 46.9% and 34%, respectively, in detecting prostate cancer. However, combining DRE and PSA increased the sensitivity of detection to 100%, specificity to 92%, and the PPV to 49% suggesting that the combination of both tests is superior to each test alone in detecting the disease 60. Similarly, a study conducted at the Soba and Omdurman teaching hospitals from 2011–2012 found that both the serum levels of early prostate cancer antigen-2 and PSA were significantly raised (compared to healthy controls) and could be used as useful prognostic and screening markers for prostate cancer. Both tests detected a considerable proportion of tumors missed by PSA alone 61. The detection of new cases of prostate cancer per year at the NCI-UG increased dramatically (from 8.1% of all cancers in 2002 to 17.2% in 2007) after the introduction of transrectal ultrasound 56. Also in metastasized cancer, bone scans using scintigraphy positively correlated with increased PSA values compared to patients with negative scans (*P* < 0.01) 62.

### Summary

According to the published data, cancer is increasing in the Sudan. Although the exact reason behind this increase is not known, it partially could be attributed to exposures to common and local carcinogens (tobacco and Toombak dipping) and to adoption of lifestyles seen in the developed countries. While the population of Sudan is moving toward cities and unhealthy lifestyles, resources, and infrastructure to prevent and treat the disease lag behind including clinical and basic research. Cancer in Sudan has very clear geographical distributions that may allow the understanding the etiological causes of cancer in that region. Breast cancer is the leading cause of death in women and more recently prostate cancer incidences and mortality ranked first in men. Most patients present with advanced stage disease that rarely amendable to treatment. This review has covered only the published data and may not accurately describe the current profile of cancer in Sudan. Thus, Sudan needs to implement a better method for tracking cancer incidences and mortality and examination of risk factors.

## Conflict of Interest

None declared.
